# Fragment-Based Approaches Identified Tecovirimat-Competitive Novel Drug Candidate for Targeting the F13 Protein of the Monkeypox Virus

**DOI:** 10.3390/v15020570

**Published:** 2023-02-19

**Authors:** Yasir Ali, Hina Imtiaz, Muhammad Mutaal Tahir, Fouzia Gul, Umair Ali Khan Saddozai, Ashfaq ur Rehman, Zhi-Guang Ren, Saadullah Khattak, Xin-Ying Ji

**Affiliations:** 1 National Center for Bioinformatics, Quaid-i-Azam University, Islamabad 45320, Pakistan; 2 Henan International Joint Laboratory for Nuclear Protein Regulation, School of Basic Medical Sciences, Henan University, Kaifeng 475004, China; 3 Tehsil Headquarter Hospital Bhera, Sargodha, Punjab 40540, Pakistan; 4 District Headquarter Hospital Faisalabad, Punjab 38000, Pakistan; 5 Department of Molecular Biology and Biochemistry, University of California Irvine, Irvine, CA 2697-3900, USA; 6 The First Affiliated Hospital, Henan University, Kaifeng 475004, China; 7 Kaifeng Key Laboratory for Infectious Diseases and Biosafety, Kaifeng 475004, China; 8 Faculty of Basic Medical Subjects, Shu-Qing Medical College of Zhengzhou, Mazhai, Erqi District, Zhengzhou 450064, China

**Keywords:** monkeypox virus, fragment-based drug design, tecovirimat, F13 protein, molecular dynamics simulation

## Abstract

Monkeypox is a serious public health issue in tropical and subtropical areas. Antivirals that target monkeypox proteins might lead to more effective and efficient therapy. The F13 protein is essential for the growth and maturation of the monkeypox virus. F13 inhibition might be a viable therapeutic target for monkeypox. The in silico fragment-based drug discovery method for developing antivirals may provide novel therapeutic options. In this study, we generated 800 compounds based on tecovirimat, an FDA-approved drug that is efficacious at nanomolar quantities against monkeypox. These compounds were evaluated to identify the most promising fragments based on binding affinity and pharmacological characteristics. The top hits from the chemical screening were docked into the active site of the F13 protein. Molecular dynamics simulations were performed on the top two probable new candidates from molecular docking. The ligand–enzyme interaction analysis revealed that the C2 ligand had lower binding free energy than the standard ligand tecovirimat. Water bridges, among other interactions, were shown to stabilize the C2 molecule. Conformational transitions and secondary structure changes in F13 protein upon C2 binding show more native three-dimensional folding of the protein. Prediction of pharmacological properties revealed that compound C2 may be promising as a drug candidate for monkeypox fever. However, additional in vitro and in vivo testing is required for validation.

## 1. Introduction

Monkeypox virus (MPXV) is a zoonotic orthopoxvirus that is transferable from animals to individuals [1]. In May 2022, a global outbreak of MPXV began [2], characterized by atypical clinical symptoms and an increased prevalence in males who engage in sexual activity with other males [3,4,5,6]. Human infection with the MPXV can cause a wide range of symptoms and disease severity, including asymptomatic or mild infection [2,3,7,8]. The fatality rate of monkeypox is lower than smallpox, ranging from 10% for clades I to 3.6% for clades IIa and IIb. Symptoms of monkeypox disease typically begin with fever, chills, back pain, weakness, swollen lymph nodes, and muscle aches. The bumps then develop into blisters, pustules, and scabs during the rash phase that follows. Skin lesions can range widely in number. The monkeypox outbreak in 2022 has been notable for its short incubation period [9], generally mild symptoms, and small, occasionally painful sores and rashes around the genitalia and anus [4,7]. Pregnant women, kids, and those with weak immune systems can all develop severe diseases.

Currently, there is no approved medication for MPXV disease. Even though many orthopoxviruses, including MPXV, variola virus, and rabbitpox, are susceptible to smallpox medications such as tecovirimat (ST-246), cidofovir, and brincidofovir [10,11,12,13]. Even if increased liver enzymes were seen in patients receiving brincidofovir for MPXV infection in 2018 and 2021 [7], the use of ST-246 has not been associated with any safety concerns, making it a suitable therapy choice for patients impacted by the ongoing outbreak.

The European Medicines Agency has approved ST-246 in Europe under “exceptional circumstances” [14]. The ST-246 is known to target the VP37/F13 protein of the F13L gene product of the vaccinia virus (VACV), which is necessary to produce extracellular viral particles. The VP37 protein of orthopoxviruses is critical for the envelopment of the intracellular mature virus with a Golgi-derived membrane to form an enveloped virus [15], which may then be released from the cell and has been shown to have an important role in the dissemination of the virus from the site of infection and viral virulence in VACV [16,17]. Tecovirimat works by inhibiting the viral envelope protein VP37, which blocks the final steps in viral maturation and release from the infected cell, thus inhibiting the spread of the virus within an infected host [18]. VP37 is highly conserved in the orthopoxviral genus. Interestingly, all 2022 MPXV genomes harbor the E353K substitution in the VP37 protein, this substitution being absent from the most recent common ancestor in clade IIb but previously identified in a single 2018 outbreak genome (MT903341). Researchers have recently linked the effectiveness of ST-246 against the MPXV at a nanomolar scale [19].

The recent reemergence of the MPXV has emphasized the importance of finding effective treatments to combat this potentially harmful pathogen and address the potential impact on public health. Unfortunately, traditional de novo drug development takes years to produce clinical candidates. Alternatively, modern drug designing techniques powered by genome-wide analysis [20,21] such as drug repurposing [22], structure-based drug design [23,24], and fragment-based drug design (FBDD) help provide more rapid solutions [25]. FBDD is frequently utilized in drug design as a potent method for discovering small molecule ligands or fragments called “hits”, which are then refined into lead compounds for therapeutic development. Given its encouraging findings, ST-246 may be utilized as a starting point for fragment-based drug discovery methodologies.

In this study, we used an advanced fragment-based drug-designing approach to target the F13 protein of the MPXV. We utilized ST-246, an FDA-approved drug, as a starting point, which offered benefits such as an established safety profile, reduced development time, an increased likelihood of success, and reduced risk. Based on computational methods, we were able to design a highly promising new compound that offers potential ST-246 competitors as therapeutic options for MPXV patients. When compared to ST-246, the proposed drug exhibited superior stability, comparable residual flexibility, favorable binding with the F13 protein, and favorable dynamic behavior. However, as well as to verify the effectiveness of recently developed drugs in vivo, more detailed studies, including animal model studies, will be required.

## 2. Methods

### 2.1. Modeling and Preparation of F13 Protein

The protein sequence of F13 from the MPXV was obtained from GenBank (URK20480.1) for this study. The AlphaFold2 online service [26], which is integrated into the ChimeraX software [27], was utilized to create the MPXV F13 protein’s (AGR36888.1) first tertiary structure. The protein preparation module in MOE was then utilized to assess the F13 protein’s structure for residues and atoms or side chains. This MOE-provided process concatenates the addition of hydrogen atoms, assigning partial charges using the MMFF94x forcefield [28], minimization of protein to a chosen gradient, the structure being 3D protonated, and the water molecules being removed. The structure was further validated by SAVES Server 6.0 [29].

### 2.2. Fragment Library Preparation 

The fragment database was created utilizing structure-guided ligand design by fragment replacement, which is critical in the drug development process. We specified precise fragments of ligand for replacement. At the designated location, it was necessary to add suitable segments that not only suited the remaining ligand’s geometric requirements but also worked well with the surrounding protein environment. This allowed for the quick development of a ligand library based on ST-246, either in scaffold hopping or bio-isosteric replacement [30]. The bioavailability score and logP-value cutoff were applied to the molecular library to minimize the number of molecules and create a database with possible bioactive fragments. The partial charge was also applied with the MOE in an MMFF94x force field, and the energy minimization methodology was employed on the fragment database using MOE version 2022.

### 2.3. Virtual Screening of Fragment Library

The MOE version 2022 software was utilized to conduct a molecular docking simulation using the fragment database against F13. The triangle matcher technique was employed for placement, London dG was used as the scoring function, and 30 times placement retention was chosen as the parameter. The remaining settings, including the type of minimization algorithm used, the convergence criteria for the optimization, and the type of nonbonded interactions to consider, were set according to the MOE version 2022 defaults. The active site from the F13 model identified by Li et al. (2022) [31] was selected as the docking region.

### 2.4. Molecular Docking via AutoDock Vina

The proposed F13 protein was constructed using the AutoDock suite and involved the removal of water molecules, the addition of polar hydrogen atoms, and the assignment of partial charges to each atom. Additionally, AutoDock tools were used to prepare any missing residues [32]. Following protein preparation, previously docked ST-246 was chosen as the control ligand and docked with F13. Based on Li et al., the active site was chosen using rectangular coordinates (2022) [31]. Grid box XYZ dimensions were set to X: −6.27702, Y: −2.48567, and Z: −8.66086, with a radius of 8.9. In order to cover the greatest possible number of amino acids in the active site, the search spacing was set at 0.55 Å. To cover all possible ligand binding positions, with a population of 300, the number of docking postures was limited to 100. After the determination of the relevant parameters, suggested ligands were docked with the F13 protein to identify possible hits and establish reliable binding poses. With Discovery Studio Visualizer [33] and PyMol software [34], the binding postures of the compound with the highest docking energies were presented.

### 2.5. Analysis of Computational Pharmacokinetics

Toxicity plays a significant role in the success of drug design and development. Advanced computational methods can be used to analyze the physiochemical properties of potential drugs, which has proven extremely helpful in understanding the mechanisms of action of these substances. The SWISS-ADME tool is a software program developed by the Swiss Federal Institute of Technology (ETH) in Zurich that is used to predict and evaluate the absorption, distribution, metabolism, and excretion (ADME) properties of small molecules. These properties can influence the safety and effectiveness of a potential drug and analyzing them is important for drug development. The SWISS-ADME tool was utilized to assess a set of ADME-related attributes for the chosen compounds [35]. This method has been particularly useful in drug development. For example, the FDA-approved drugs Vemurafenib and Nivolumab have been shown to have favorable ADME properties as predicted by the SWISS-ADME tool.

### 2.6. Explicit Solvent Molecular Dynamics Simulation

The Schrödinger software’s Desmond program (version 2021-2) [36] was used to perform molecular dynamics (MD) simulations on protein conformational changes, as previously implemented in our research [24,37,38,39]. The OPLS4 (optimized potentials for liquid simulation) force field was utilized. To simulate the MPXV-F13 protein complex, a triclinic periodic boundary box was created with dimensions of 10 Å in each direction and solvated using an explicit solvation model (Monte Carlo equilibrated TIP3P) [40]. Lennard-Jones interactions (cut-off = 10) and the SHAKE algorithm [41] were utilized to regulate covalent bond mobility, including hydrogen bonds, and 0.15 M Na+Cl was added to neutralize the system during solvation. The protein models were energy-minimized using an NPT ensemble until a gradient threshold of 25 kcal/mol/Å was reached at a temperature of 300 K and pressure of 1 bar. Each system underwent independent MD runs, and the trajectory was recorded at a time interval of 50 ps. The Particle Mesh Ewald method was used to account for long-range coulombic interactions, while the RESPA integrator regulated all covalent bonds involving hydrogen atoms [42]. The inner time step was set at 2 fs throughout the simulation. For short-range electrostatic interactions, a value of 9.0 was chosen, and uniform density was used for long-range van der Waals interactions. A 12 ps Nosé–Hoover thermostat [43] was utilized at a temperature of 300 K and pressure of 01 atm, and the Martyna–Tobias–Klein barostat technique with a 12 ps relaxation period was utilized [44]. The stability of each system was analyzed using MD simulation trajectories and metrics such as RMSD, RMSF, Rg, H-bond occupancies, and SSE using Schrödinger 2021-2 [36].

### 2.7. Essential Dynamics Analysis via Principal Components

The internal collective movements of the F13 protein for the bound ligands ST-246, C2, and C3 were investigated by computing the positional covariance matrix for the backbone Cα atoms of the residues. All trajectories were realigned on the protein Cα atoms of the initial structure of their respective complexes (Cα atoms for ST-246, C2, and C3). All frames across trajectories were considered for each simulation. The eigenvalues and eigenvectors were extracted from the 400 ns long MD trajectories using the ProDy tool [45], and the eigenvectors were analyzed and plotted using Python scripts and Numpy [46] and Matplotlib libraries [47]. The estimated ten eigenvectors (as a function of percentage) were projected on a line graph with markers for comparison purposes.

## 3. Results

### 3.1. Preparation of F13 Protein 

**Figure 1:**
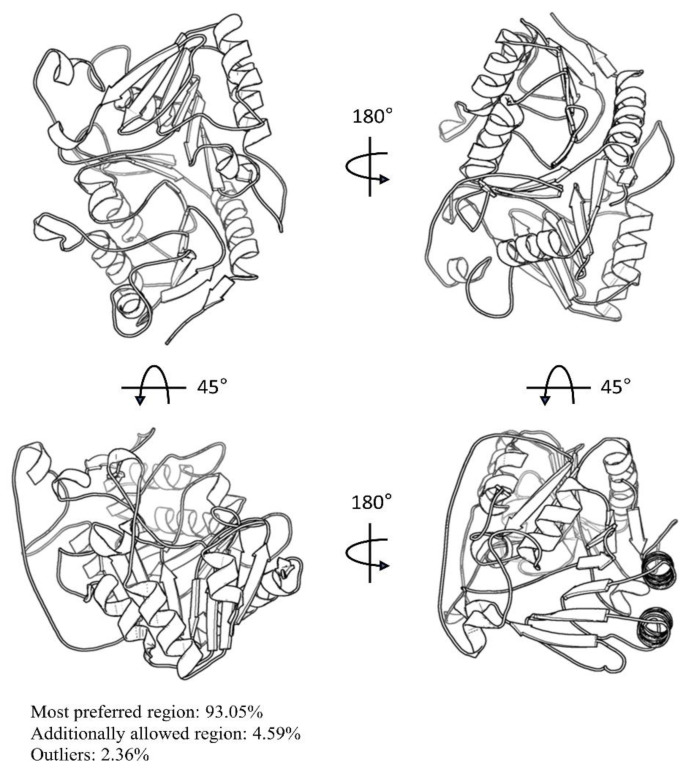
The predicted structure of MPXV-F13 protein using the AlphaFold, embedded in the ChimeraX program.

The protein sequence for the F13 protein from Genbank (URK20480.1) was downloaded. Next, the conserved domains were annotated by the National Center for Biotechnology Information (NCBI) Conserved Domain Database (CDD). The sequence consistency of F13 between the MPXV and variola virus was conservative, ranging from 97.58% to 99.73%. The projected F13 protein structure of AlphaFold is comparable to the F13 protein of other pox viruses. The predicted local distance difference test (pLDDT) score was 92.33, suggesting that the F13 model had extremely high confidence. SAVES v6.0 was used to verify the model by examining its Ramachandran plot and establishing the 3D model’s consistency with its sequence. The distribution of ϕ and ψ angles of amino acid residues derived from Ramachandran plots revealed that 93.05% of the areas were in the most preferred region, 4.59% were in the extra permitted region, and just 2.36% were outliers (Figure 1). The more it fell in the most favorable zone, the more exactly the structure was replicated, and hence, the F13 model was very trustworthy. The Z-score of atoms (0.2), Z-score RMS (0.5), and a 3D–2D score of the residues (84.95% ≥ 0.2) suggested that the F13 structure was well resolved. We used quick prep in MOE 2022 to minimize and optimize the F13 protein using Amber10: EHT force field. The F13 structure was minimized when the RMS gradient was 0.001 kcal/mol/Å^2^.

### 3.2. Fragment-Based Compounds Library Development Using ST-246 Molecular Properties

**Figure 2:**
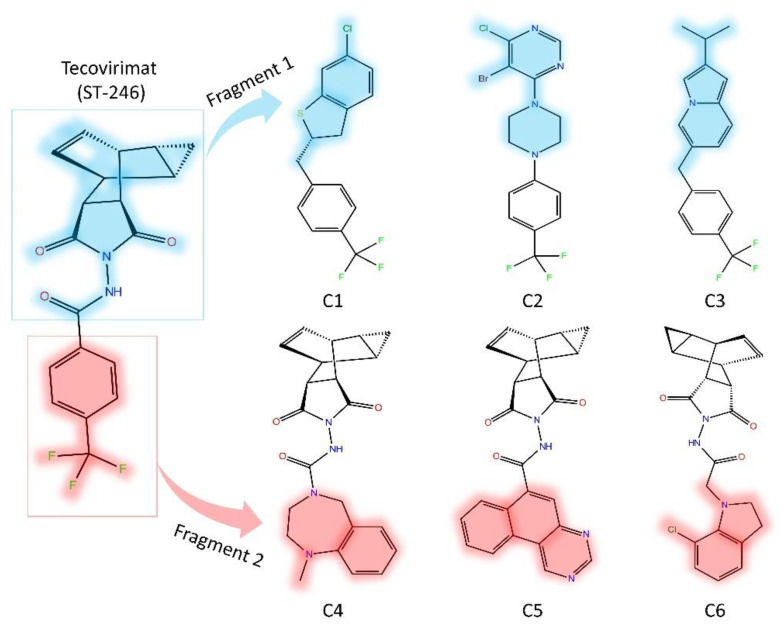
The identified top three hits of each fragment after virtual screening against library developed using fragment replacement.

The biogenic database of ST-246 competitive novel drug candidates was developed using two fragments that cover the whole ST-246 structure (Figure 2). To screen and generate the fragment libraries, we employed LogP and bioavailability scores. To limit the number of most probable fragments, the Lipinski rule was used. Furthermore, the Lipinski rule aided in the creation of libraries that only include drug-like features and simple compounds that are efficient and potentially bioactive. In principle, the Lipinski rule would lower the molecule’s complexity in a fragment library that meets the following criteria: There can be no more than 5 hydrogen bond donors (OH and NH groups); there can be no more than ten hydrogen bond acceptors (notably N and O) and a molecular weight of less than 500 g/mol and a log P partition coefficient less than 5 (Table 1). 

### 3.3. Virtual Screening and Selection of Top Hits

The virtual screening resulted in tabulated binding affinities of all the compounds. We picked the top three compounds from each fragment and subjected them to molecular docking against the MPXV-F13 protein. Molecular docking is a technique for predicting and determining the optimal interaction between a ligand and a receptor (e.g., enzyme or protein). 

### 3.4. Molecular Docking Analysis

**Figure 3:**
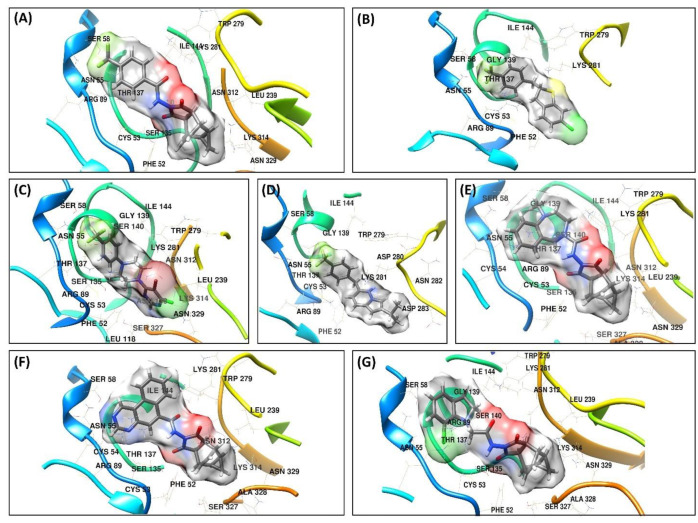
The three-dimensional interactions and orientation of (**A**) ST246 and newly identified compounds from fragment 1, i.e., (**B**) C1 (**C**) C2 and (**D**) C3, (**E**) C4 (**F**) C5, and (**G**) C6.

Vina binding energies were obtained from the result of molecular docking that was used to predict favorable ligand binding conformation with F13 protein. In this study, we docked top hits toward the MPXV-F13 protein to obtain the binding affinities and their binding interaction (Figure 3). The binding energy along with the detail of the residues that participated in hydrogen bonds and hydrophobic interactions are tabulated in Table 2. 

The ST-246, taken as a control in this study, showed a binding energy of −8.3 kcal/mol (Figure 3A). The three hydrogen bonds were observed with the protein. The residues Asn55, Ser58, and Arg89 formed the hydrogen bond with the trifluoromethyl-benzene, whereas Asn55 and Arg89 were engaged in more than one type of interactions, i.e., Halogen and Pi-Cation interactions, respectively. The residues Phe52 and Leu118 formed a Pi-Alkyl interaction with the (1R,2S,4R,5S)-tricyclo [3.2.2.02,4]non-6-ene part of the compound. The C1 exhibited a binding affinity of −8.9 kcal/mol. The Phe52 formed Pi-Pi T-shaped interaction with chlorobenzene (Figure 3B). The tetrahydrothiophene part of the compound formed Alkyl interaction with Lys281. The trifluoromethyl-benzene formed most of the interactions and anchored the compound in the binding cavity of the protein. Arg89 and Asn55 established hydrogen bonds as well as halogen interactions with Florine. In addition to this, the residues Arg89 and Cys53 formed Pi-Sulfur and Pi-Cation interactions with the benzene ring of trifluoromethyl-benzene, respectively. The highest binding energy of −9.2 kcal/mol was exhibited by C2. The 5-bromo-4-chloropyrimidine group of the compound engaged Asn312 and Ser327 in conventional hydrogen bond and carbon-hydrogen bond, respectively, whereas Leu239 and Lys314 engaged in Pi-Cation and Alkyl interactions (Figure 3C). The residue Ser135 formed a carbon-hydrogen bond with the Piperazine group of the compound. The three hydrogens were observed between the trifluoromethyl-benzene group and Arg89, Asn55, and Thr137, while Cys53 formed a Pi-Sulfur bond. C3 showed a binding energy of −7.03 kcal/mol. Two hydrogen bonds formed between Arg89 and Thr137 and trifluoromethyl-benzene. In addition to this, trifluoromethyl-benzene also formed Pi-Cation and Pi-Sulfur bonds with Cys53 and Arg89, respectively. Lys281 formed one carbon-hydrogen bond and two Pi-Cation bonds between the 2-isopropylinodolizine group of compounds (Figure 3D).

The binding energy of −7.1 kcal/mol was recorded for the C4 compound (Figure 3E). The Cys53 and Arg89 were involved in the Pi-Sulfur and Pi-Cation interaction with the benzene ring of the compound, respectively, whereas the 1,4-diazepane formed a carbon-hydrogen bond with Ser58. Furthermore, the residue Phe52 formed Pi-Alkyl interactions with the (1R,2S,4R,5S)-tricyclo[3.2.2.02,4]non-6-ene group of the compound. A −6.9 kcal/mol binding energy was observed for the C5 compound. The benzo[*f*]quinazoline group on the compound formed the hydrogen bond and Pi-Alkyl with the residues Asn55 and Cys53, respectively, whereas Arg89 formed two Pi-Cation interactions, and Lys281 formed one Pi-Cation with the benzo[*f*]quinazoline. The residues Phe52 and Leu118 established Alkyl interaction with the (3aR,4R,4aR,5aS,6S,6aS)-4,4a,5,5a,6,6a-hexahydro-4,6-ethenocyclopropa[*f*]isoindole-1,3(2H,3aH)-dione group of the compound (Figure 3F). The compound C6 exhibited the highest binding energy of -6.6 kcal/mol among all other compounds (Figure 3G). Chlorobenzene formed a carbon-hydrogen bond with Arg89. Two Pi-Alkyl interactions were also observed between the residues Phe52 and Leu118 with the (1R,2S,4R,5S)-tricyclo[3.2.2.02,4]non-6-ene group. In addition to this, Cys53 also formed a conventional hydrogen bond.

### 3.5. Computational Pharmacokinetics

The top-performing compounds were subjected to pharmacokinetics studies. The SWISS-ADME, an online server, was utilized to perform a Lipinski rule of five analysis on the top six compounds and ST-246. We noticed that none of the compounds violated any of the Lipinski rules and all the properties were well within the optimum range as per the Lipinski rule of five. The results of the Lipinski rule of five are summarized in Table 3.

Absorption, distribution, metabolism, and excretion (ADME) are pharmacokinetic processes that characterize a drug’s overall disposition. ADME analysis evaluation was carried out to determine whether the compounds designed by structure-based fragment replacement had a favorable ADME, which is the primary method for determining drug molecule effectiveness. To determine the aqueous solubility, three methods, ESOL, ALI logS, and SILICOS-IT logS, were employed [35] Results revealed that compound C2 has comparable results when compared to the FDA-approved Tecovirinat (ST246) drug. Both drugs have blood–brain barrier permeability with high Gastrointestinal (GI) absorption. Additionally, both drugs have the properties to be used as CYP2C19 and CYP3A4 inhibitors. The ADME properties along with their predicted attributes are tabulated in Table 4.

### 3.6. F13 Protein Stability upon Binding

**Figure 4:**
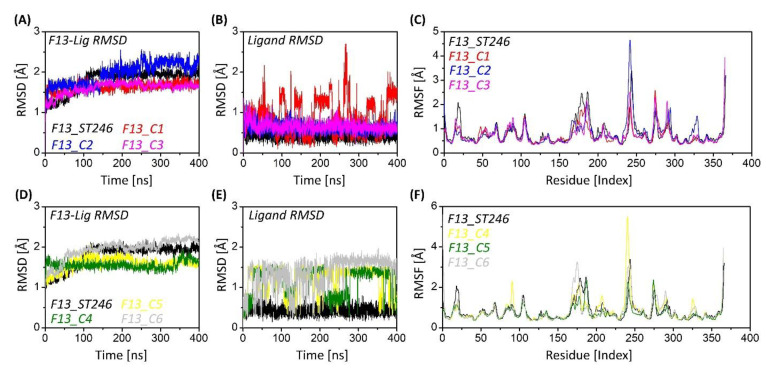
Root mean square deviation and fluctuation (RMSD/F) of individual ligands and Cα of protein residues. (**A**) Compounds generated from fragment 1 in complex with the F13 protein. (**B**) individual RMSDs of ST-246, C1, C2, and C3. (**C**) RMSF plots analysis of fragment 1-based compounds. (**D**) Compounds generated from fragment 2 in complex with the F13 protein, and (**E**) individual RMSDs of ST-246, C4, C5, and C6, and (**F**) square fluctuations of fragment 2-based compounds after 400 ns of simulation.

The ST-246 docked to F13 was considered the control as its activity against the MPXV-F13 is already reported. The ST-246 exhibited the average RMSD of 1.79 Å when in complex with F13 protein (Figure 4A,C) compared to 1.64 Å, 1.95 Å, 1.60 Å, 1.58 Å, 1.55 Å, and 1.94 Å of C1, C2, C3, C4, C5, and C6. The control system was stable with some minor fluctuations at the start of the simulation until it converged at 1.90 Å. The RMSD of ST-246 with respect to its initial position was quite low (0.50 Å), which shows its firm binding with the active site of MPXV-F13 protein. The noted RMSD of ST-246 was lower than all other ligands and comparable to C2 (0.62 Å) and C3 (0.65). (Figure 4B). The compounds C2 and C3 showed a behavior like ST-246. The RMSD plot showed compactness. This shows that the control and C2 and C3 ligands were very well anchored in the binding pocket and F13 and established stable interactions. Additionally, all the other compounds showed an increasing RMSD trend, which is also observed in the RMSD plots (Figure 4B,D); however, going forward, all the compounds escaped the active site, though in some instances, compounds C3 and C4 showed binding with the active site. Still, it was only for a while, and they left the active site soon after. These results showed that out of the six identified compounds, only compounds C2 and C3 showed satisfactory results that could be ST-246-competitive inhibitors of MPXV-F13 protein.

### 3.7. F13-ST246 Complex and F13-C2 Complex Have Comparable Flexibility

It is necessary to evaluate the mobility of the atoms of residues and the structural integrity of the system. Root mean square fluctuation (RMSF) analyzed the flexibility of protein residues (Figure 4C,F) [48]. Among all complexes, the maximum mean RMSF of 0.83 Å was observed for the C6-bound F13 protein, which was also higher than that of the ST246-F13 complex. The mean RMSF of 0.81 Å each was noted for the ST-246 and C2-F13 complex. The minimum mean RMSF of 0.82 Å was exhibited by the C4-F13 complex. The C1 and C3-F13 showed a mean RMSF of 0.73 Å each, while the C5 bound F13 complex showed the least average RMSF of 0.71 Å. The coils and the loops were the most fluctuating regions as they are the connection between alpha-helices and beta-strands. These sheets and helices are often oriented throughout the simulation to allow for ligand interactions and ensure the ligand remains inside the active site. This demonstrates the technique of adapting the ligand’s active site preferences and the ligand’s approach of aiming for a stable binding at last. Moreover, the highest fluctuating residue in the ST-246 complex was residue Ser250 (3.50 Å), the C1 complex was Lys371 (3.43 Å), and the C2 complex was Asp248 (4.83 Å), whereas C3, C4, C5, and C6 complexes were residues Lys371(3.94 Å), Val247 (5.763 Å), Lys371 (3.67 Å), and Ser6 (5.83 Å), respectively.

### 3.8. F13-ST246 and F13-C2 Complexes Have Inline Solvent Accessibility and Compactness

**Figure 5:**
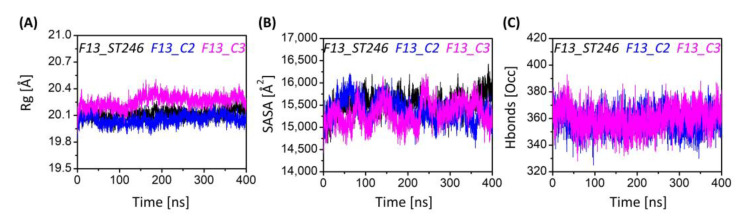
The plots show the results of (**A**) radius of gyration (Rg), (**B**) solvent accessible surface area (SASA), and (**C**) hydrogen bond occupancy analysis during the simulation time of 400 ns per system. The line graph has been enriched with color-coded legends for each graph.

The information regarding the system’s equilibrium is measured using the radius of gyration (Rg). The Rg tells us about the system compactness, either increasing or decreasing upon perturbation [49] The Rg is inversely linked with the compactness of the protein as at higher values the atoms will relax, whereas at lower values the atoms will observe tight packing. The mean Rg of the ST-246, C2, and C3 systems was recorded as 20.11 ± 0.05 Å, 20.25 ± 0.06 Å, and 20.05 ± 0.06 Å, respectively (Figure 5A). The lowest Rg value was exhibited by the C3 system, while the higher Rg trend of C2 after 180 ns depicts the shift of the complex from one state to another, which depicts that the structural changes occurred to attain stability that remained until the end of the simulation. During the simulation period, the complexes demonstrated a time-dependent increase and decrease in SASA (Figure 5B), which is exactly in line with the gyration plot. The average SASA values for ST246-F13, C2-F13, and C3-F13 were 15,521 ± 241 Å^2^, 15,311 ± 204 Å^2^, and 15,383 ± 256 Å^2^, respectively. These values show that the higher SASA was exhibited by ST246-F13, whereas the lower was exhibited by C2-F13. The least solvent-accessible surface area, 14,637 Å^2^, 14,486 Å^2^, and 14,406 Å^2^, was noticed at the start of the simulation for the three (ST246-F13, C2-F13, and C3-F13) systems, respectively. The maximum exposed surface was noted at 390 ns, 250 ns, and 6 ns, respectively, with an area of 16,426 Å^2^, 16,069 Å^2^, and 16,214 Å^2^.

### 3.9. F13 Protein in Complex with ST246 & C2 Enhance the Intraprotein hb Population

Hydrogen bonds play a significant role in ligand binding to receptors with both high specificity and affinity [50,51]. For that reason, the hydrogen bond occupancy of all the complexes was examined throughout the simulation. The hydrogen bond occupancy of all complexes is depicted in Figure 5C. The average hydrogen bond occupancy for ST246, C2, and C3 in complex with F13 protein was 359, 358, and 360. The hydrogen bond occupancy exhibited by all complexes was noted in the range 325–393. The higher denser hydrogen bonds were possessed by C3-F13, which is why the complex has been compact with no transition to another state. Moreover, in terms of hydrogen bonding, all the complexes are quite consistent with one another. It can be concluded that during the simulation, C3-F13 demonstrated the most hydrogen bonding.

### 3.10. Exploring Transitional Conformations upon Binding of F13 Protein to ST246 & C2

**Figure 6:**
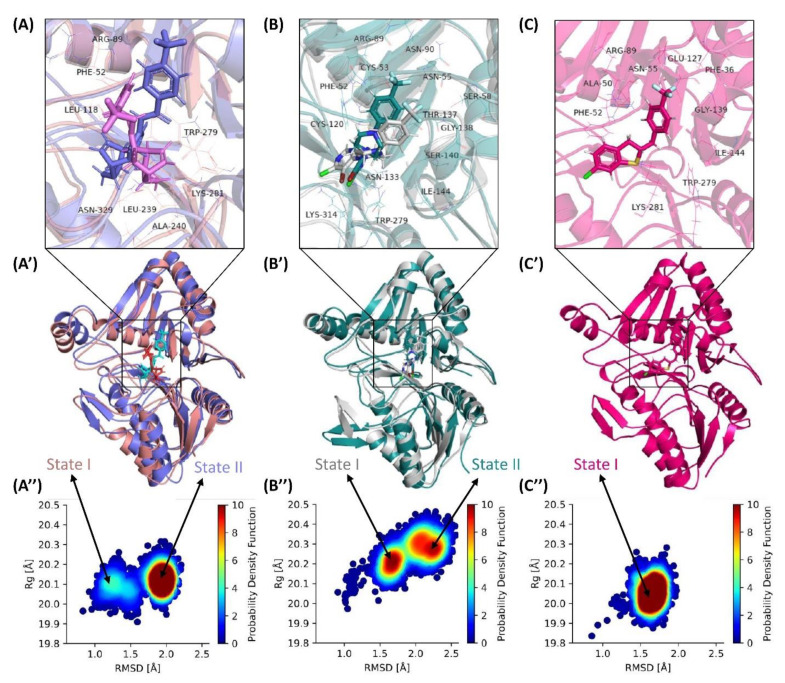
Probability distribution of root means square deviation (RMSD) and radius of gyration in 2D space. The data from molecular dynamics (MD) runs were integrated into each model, i.e., (**A”**) ST-246 (**B”**) C2, and (**C”**) C3. (**A’**–**C’**) show the most preferred orientation of protein and ligand in state I and state II. (**A**–**C**) show the active site of view of ST-246, C2, and C3 in the active site of MPXV-F13 protein, respectively.

To further elucidate the compactness and measure the sizes of our simulated F13-bound ST-246, C2, and C3 complexes, we plotted the distribution with coordinates of Rg and RMSD (Figure 6”) for the whole trajectories. For ST246 simulations, the distribution of structural compactness (Figure 6A”) ranged from 19.9 Å to 23.3 Å for Rg, whereas the RMSD distribution ranged from 0.7 Å to 2.3 Å. It also showed relatively two most preferred conformations of the protein, one with gyration at 20.1 Å and RMSD with 1.25 Å and a second with Rg ranging around 20.1 Å and RMSD at 2.0 Å, with the latter more favored. This indicates that though ST246-bound F13 protein is changing its state, a long-range simulation is required to know the phenomenon of transition from one state to another. In contrast, compound C2 exhibited highly inline but more prominent state I and State II. The first one ranged from 20.2 Å and RMSD with 1.6 Å and was more prominent compared to ST-246. Another distribution of high probability represented a different conformation ranging from 20.2 Å to 20.3 Å for Rg with RMSD of 2.1 Å to 2.4 Å. This observation indicates a larger populated structural distribution and diverse conformations. However, simulations of ST-246 showed wider dispersed state I, while Compound C2 create more ordered and prominent conformational states. While Compound C3 exhibited only a single state ranging from Rg of 20.05 Å to 20.1 Å and RMSD of 1.5 Å to 1.7 Å, which is highly compact conformation with no transition to the later state that was noted in the Control ST-246 (Figure 6C”).

### 3.11. C2 Binding to F13 Offer Conformational Stability

**Figure 7:**
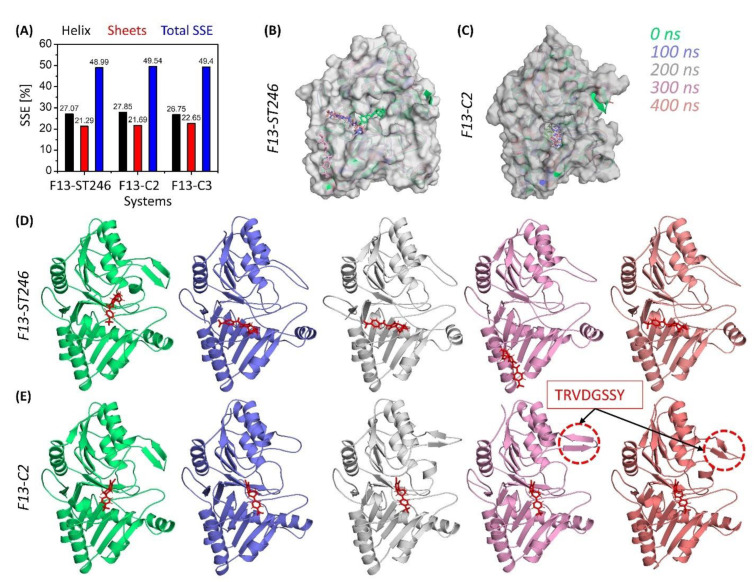
Secondary structure and conformational analysis of ST-246 and new candidate drug C2 against MPXV-F13 protein. (**A**) Percentage secondary structure elements of ST246, C2, and C3-bound to F13 protein of MPXV. (**B**) ST246 propagation throughout the trajectory. (**C**) C2 bound MPXV at regular intervals of time. (**D**) Comparison of secondary structures of ST246-bound MPXV-F13 protein, and (**E**) comparison of secondary structures of C2-bound MPXV-F13 protein using cartoon representation.

During simulation, protein secondary structural elements (SSE) such as alpha-helices and beta-strands were examined. Analysis of the secondary structural elements (SSE) that contribute to protein stability revealed that all proteins maintained an average of 48–49% SSE, with helices dominating over sheets. The SSE of all complexes exhibited very minor differences. Typically, the terminal regions (N-terminal and C-terminal) of proteins fluctuate more than any other portion of the protein. Secondary structure elements such as α-helices and β-strands deviated less (Figure 7). The important difference that was noticed in the behavior of F13 protein upon binding to ST246 and C2 was the making of the beta-hairpin (res no 245-252; TRVDGSSY) when bound to C2. This change was rarely observed for ST246 and was not observed for C3. The formation of a beta-hairpin is the sign of the nucleation site, which is the first step in the formation of either a new thermodynamic phase or a new structure via self-organization. This also supports the transition of C2-bound F13 protein to a more native three-dimensional structure as shown in the probability density function.

### 3.12. Binding Energy Calculation Shows a Favorable Profile for F13-C2 Complex

**Figure 8:**
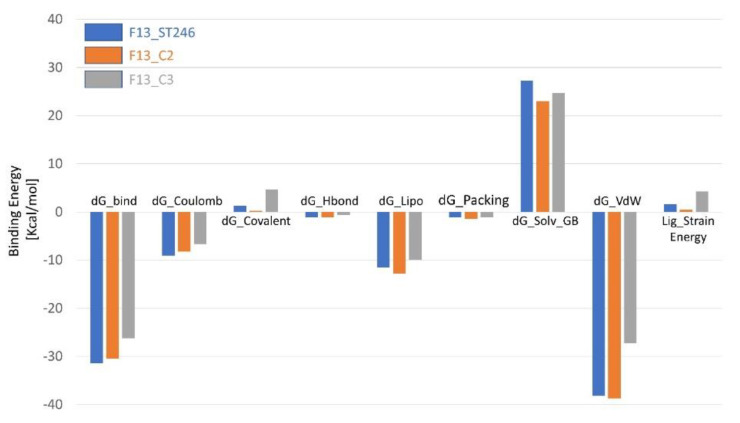
The molecular mechanics, generalized born, and surface area solvation energies of ST246 (blue), C2 (orange), and C3 (gray) in complex with F13 protein of MPXV.

The simulation results of the binding energies of three complexes (F13_ST246 as control and C2 and C3 as identified drug candidates) against the monkeypox virus’s F13 protein were calculated by the PRIME MMGBSA module after 400 ns simulation time. Among the top contributing energies, dG_Bind, dG_Coulomb, and dG_vdW showed the greatest differences among the complexes. The lowest dG_Bind was observed for F13_ST246, with a value of −31.38 kcal/mol, followed by F13_C2 (−30.49 kcal/mol) and F13_C3, with a value of −26.24 kcal/mol. F13_ST246 also showed the least dG Coulomb value of −9.07 kcal/mol, while F13_C2 has the comparable binding coulomb energy of −8.26 kcal/mol, followed by F13_C3, which had the highest binding coulomb energy of −6.68 kcal/mol. The dG lipophilicity of F13_C2 has the most favorable results of −12.75 kcal/mol, which in turn, reduced the solvation energy among all the compounds (Figure 8). Additionally, favorable van der Waals energies mainly contributed to the binding energies of all the compounds. The dG Covalent, dG Hydrogen bond, and dG Packing values were relatively similar among the three complexes, with slight variations. The lowest ligand strain energy also supported the favorable binding energies of the C2 compound. Based on the binding energy analysis, it can be concluded that F13_C2 appears to be the best drug candidate when compared to the control drug ST-246, as it had the comparable dG Bind and lowest dG lipophilicity and dG Solvation values, indicating a stronger interaction between the compound and F13. The relatively low dG van der Waal value suggests a favorable interaction between the compound and solvent, making it a more stable complex overall.

### 3.13. Dimensionality Reduction via Principal Components Analysis (PCA)

**Figure 9:**
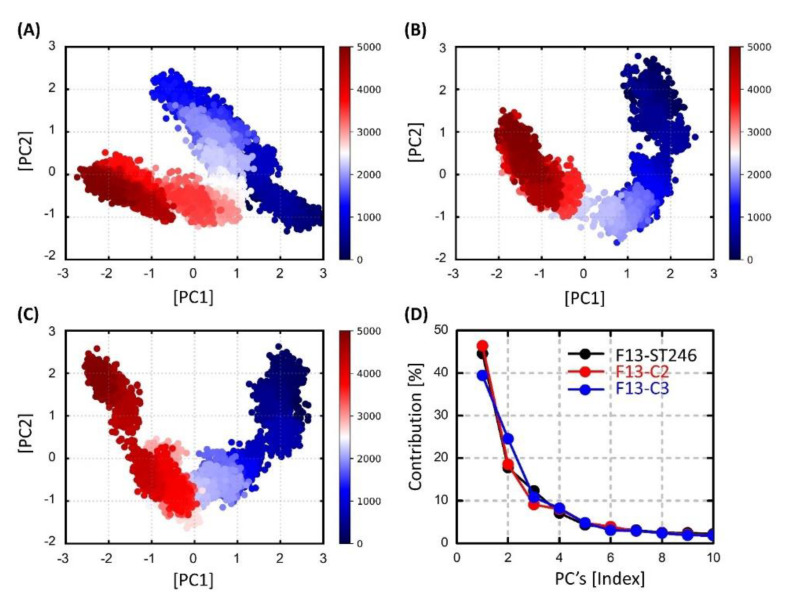
Scatter plot generated for the first two principal components via python scripts using built-in matplotlib and NumPy libraries for (**A**) ST246-bound MPXV F13 protein, (**B**) F13-C2 complex, and (**C**) C3-bound F13 protein along with (**D**) the percentage contributions of 10 principal components.

The dimensionality reduction allowed us to analyze the prominent collective dynamics of the bound F13 protein. The F13-ST246 complex initial motions were spread out as compared to the C2 complex, which exhibited relatively clearly two different states (Figure 9). However, both complexes were transformed into a second state after half of the simulation time. It is noticed that during this phase both complexes are much more compact, and no abrupt changes were noticed. Alternatively, the C3-F13 complex was found highly unstable, making different clusters that are not dense, showing that the compound was not stable at any stage during the simulation. In line with the PCA results, the first principal component contributed 44.59%, 46.40%, and 39.45% to the total collective motions, which is much lower compared to the other two complexes.

## 4. Discussion

MPXV is a zoonotic virus that causes an infection similar to smallpox, with frequent complications and sequelae in susceptible populations. Since its discovery in the Democratic Republic of the Congo in 1972 [52], MPXV infections have been documented regularly in East Africa [1] and West Africa [53]. When an epidemic of MPXV occurred in the United States in 2003, the risk of infection increased [54]. Wild rodents brought in from Ghana affected prairie dogs, which subsequently served as a reservoir for human illness. These biological concerns provide an additional reason to seek the development of novel MPXV vaccines and treatments.

In 2022, the contagious viral disease human monkeypox spread quickly, triggering a worldwide health emergency [55]. The MPXV genomes have been widely studied and published, but very little is known about the encoded proteome of the virus. Other than computational models, no experimental protein structures of the newly emerged MPXV strains have been published. Minasov et al. 2022 solved the X-ray structure of the MPXV protein A42R, the first MPXV-encoded protein, at 1.52 Å resolution [56]. In (May) 2022, Frenois-Veyrat et al. identified and sequenced a virus from the first clinical MPXV case detected in France. They found that tecovirimat (ST-246), a drug licensed by the US Food and Drug Administration, is effective against this isolate in vitro at nanomolar quantities. This was the first instance of the in vitro association of ST-246 effectiveness against MPXV and the second instance of ST-246 recommendation for the current human MPXV epidemic [19]. Previously, Li et al., 2022, also reported the potential activity of ST-246 against F13 protein against human MPXV using biocomputational resources [31].

New therapeutic development is challenging, time-consuming, and costly. In the event of an eradicated disease (such as smallpox), the issue is much more complicated and does not seem to be worthy of further pharmaceutical company attention owing to a poor cost–benefit ratio. Thus, the recent FDA approval of ST-246 to treat a possible MPXV reemergence is a significant step forward in this regard. Efforts to create novel antivirals, however, are required to anticipate the advent of resistant virus strains. This problem may be addressed by developing methods based on several treatments or by combining medications with diverse targets and modes of action. The WHO advisory committee on MPXV research advised that research on medicines with potential antiviral activity be continued in suitable animal models employing surrogate MPXV viruses. To round out the present pharmacopeia, compounds with high effectiveness and low toxicity should be studied. ST-246 and cidofovir, as previously indicated, are in advanced development and may be future possibilities for the treatment of MPXV infections [19]. Since orthopoxviruses create extracellular virion, which is essential for rapid cell-to-cell spread and long-distance dissemination [57], F13, which is encoded by poxvirus, is essential for the generation and infectivity of extracellular virion [58], making its inhibition a favorable target for drug designing. Recent research shows that ST-246, a wide inhibitor of orthopoxvirus F13, might be used to combat reemerging monkeypox [7].

The reemergence of the MPXV has highlighted the need for effective treatments to combat this pathogen and address its potential impact on public health. In this scenario, computational approaches can significantly reduce the time and cost to develop new modulators or antivirals to combat this disease [59]. In this study, we employed advanced fragment-based drug design approaches and utilized the FDA-approved drug ST-246 as a starting point to target the active site of the F13 protein of the MPXV. By applying computational drug design techniques, we were able to successfully design a new compound, C2, with improved chemical properties compared to ST-246. Specifically, C2 exhibits superior stability, comparable residual flexibility, and favorable binding with the F13 protein. The ability of C2 to promote the formation of a beta-hairpin in the F13 protein is also of particular interest. Beta-hairpins are essential structural elements in proteins that contribute to their stability and function. In the context of the F13 protein, the formation of a beta-hairpin at this specific site may be linked to the nucleation of a specific site on the protein surface, such as a binding site for another molecule. Further investigation into the precise role of this beta-hairpin in the function of the F13 protein may provide valuable insight into the biology of the MPXV and aid in the development of future treatment strategies.

Overall, our study demonstrates the potential of computational techniques for the successful treatment of emerging viruses such as MPXV. Further testing is needed to fully evaluate the safety, dosage, and efficacy of C2 in clinical trials in animal models and humans to establish the efficacy of ST-246 competitive novel drugs in vivo. However, the promising results of this study suggest that C2 (5-bromo-4-chloro-6-(4-(4-(trifluoromethyl)phenyl)piperazin-1-yl)pyrimidine) may be a valuable addition to the arsenal of antiviral therapies for the treatment of monkeypox and other orthopoxviruses.

## 5. Conclusions

The current study identified a total of six candidate compounds based on the fragment replacement of the FDA-approved drug (ST-246) against the MPXV-F13 protein. A molecular docking study revealed that the binding energy (∆G) of these ligands was lower than the negative control. Particularly, C2 and C3 showed the best potential (∆G −8.9, −9.2 kcal/mol) among all the identified compounds compared with standard drug ST-246 (−8.3 kcal/mol). When compared to the standard drug, computational toxicity and drug-likeness testing yielded drug-likeness of the compounds. Extending our results further, molecular dynamics, binding energies, and essential dynamics results delineated that among all the identified compounds, C2 showed the best potential against the target and may inhibit monkey pox’s F13 protein activity. Finally, it is concluded that the C2 compound can potentially inhibit the viral envelope protein by blocking the viral maturation and subsequently its release from the infected cell, thus inhibiting the spread of the virus within an infected host. However, additional in vitro and in vivo experiments are required to verify its inhibitory effectiveness and bioactivity against F13 protein as an MPX treatment.

## Figures and Tables

**Table 1:** The molecular properties of ST-246 that were considered for fragment replacement in addition to the F13-bound ST-246 environment.

Property	Value	Property	Value
Number of atoms	27	Lipinski Hyd. bond donor	1
Number of heteroatoms	8	*TPSA*	66.48
HBA	3	Lipinski Hyd. bond acceptors	5
Aromatic Carbocycles	1	Rings	6
LogP	2.403	Mol Wt	372.103
RB	2	Labute ASA	151.724
Aromatic Heterocycles	0	Aromatic rings	1
No. of Aliphatic Carbocycles	4		

Remarks: No., number; HBA, hydrogen bond acceptor; RB, rotatable bonds; Hyd, hydrogen; TPSA, topological polar surface area; Mol Wt, molecular weight.

**Table 2:** Molecular docking results of top hits with MPXV-F13 protein. The results are based on binding affinities from AutoDock Vina.

Compound	Binding Energy (Kcal/mol)	Hydrogen Bonds	Hydrophobic Interactions
ST-246	−8.3	Asn55, Arg89, Ser58	Asn55 Arg89, Cys53, Phe52, Leu118
C1	−8.9	Arg89, Asn55	Phe52, Lys281, Cys53, Arg89,
C2	−9.2	Arg89, Asn55, Asn312, Thr137, Ser135, Ser327	Cys53, Leu239, Lys314, Cys53
C3	−7.03	Arg89, Thr137, Lys281	Cys53, Arg89, Lys281
C4	−7.1	Ser58	Cys53, Arg89, Phe52
C5	−6.9	Asn55	Cys53, Phe52, Arg89, Leu118, Lys281
C6	−6.6	Cys53, Arg89	Leu118, Phe52

**Table 3:** The Lipinski rule of five is applied to the top three compounds of each fragment.

Compounds	MW	RB	HBA	HBD	TPSA	Log P
ST-246	376.33	4	6	1	66.48	2.65
C1	328.78	3	3	0	25.3	5.64
C2	421.64	3	5	0	32.26	3.94
C3	317.35	4	3	0	4.41	5.47
C4	392.45	3	3	1	72.96	1.38
C5	410.42	3	5	1	92.26	2.23
C6	397.85	4	3	1	69.72	2.17

Remarks: MW, molecular weight; HBA, hydrogen bond acceptor; HBD, hydrogen bond donor; RB, rotatable bond; TPSA, total polar surface area; MW > 500, H.B.D < 5, H.B.A < 10, R.B < 10, 140 < TPSA > 0, Log P < 5.

**Table 4:** The ADME properties of the compounds.

Compounds	Solubility			BBB Permeant	Pharmacokinetics	
	Log S (ESOL)	Log S (Ali)	Log S (SILICOS-IT)		GI Absorption	CYP Enzymes Inhibitors
ST-246	Soluble	Soluble	Soluble	Yes	High	CYP2C19 and CYP3A4 inhibitor
C1	Moderately soluble	Poorly soluble	Poorly soluble	No	Low	CYP1A2, CYP2C9, and CYP2D6 inhibitor
C2	Moderately soluble	Moderately soluble	Moderately soluble	Yes	High	CYP1A2, CYP2C19, and CYP2C9 inhibitor
C3	Poorly soluble	Poorly soluble	Poorly soluble	No	Low	CYP1A2, CYP2C19, and CYP2D6 inhibitor
C4	Soluble	Soluble	Soluble	No	High	CYP3A4 inhibitor
C5	Moderately soluble	Moderately soluble	Moderately soluble	No	High	CYP1A2 inhibitor
C6	Soluble	Soluble	Soluble	No	High	CYP2C19, CYP2D6, and CYP3A4 inhibitor

## Data Availability

The data presented in this study are available in this article.
